# Paper Analytic Device to Detect the Presence of Four Chemotherapy Drugs

**DOI:** 10.1200/JGO.18.00198

**Published:** 2018-12-27

**Authors:** Madeline Smith, Ayenew Ashenef, Marya Lieberman

**Affiliations:** **Madeline Smith** and **Marya Lieberman**, University of Notre Dame, Notre Dame, IN; and **Ayenew Ashenef**, Addis Ababa University, Addis Ababa, Ethiopia.

## Abstract

A paper analytic device, the chemoPAD, was developed and validated to visually detect methotrexate, doxorubicin, cisplatin, and oxaliplatin at concentrations commonly found in injectable dosage forms. By testing residual solution after patient treatment, the chemoPAD can be used to monitor drug quality without restriction of patient access to medication. The chemoPAD is produced by wax printing on Ahlstrom paper to create separate reaction areas and deposits small amounts of chemicals to create color changes in response to different active pharmaceutical ingredients (APIs). This creates a unique color bar code to identify each medication. Internal validation studies show that the chemoPAD has excellent sensitivity and specificity to differentiate between samples of 100% and 0% API, which is the distinction relevant to the majority of reported falsified chemotherapy cases. The platinum-containing drugs, cisplatin and oxaliplatin, can be detected semiquantitatively. The cards can be read either visually by comparison with a standard image or by using computer image analysis. Dosage forms were collected from the Ethiopian health care system and analyzed with the chemoPAD followed by high-performance liquid chromatography. A substandard sample was discovered and reported to the Ethiopian Food Medicine and Healthcare Administration and Control Authority.

## INTRODUCTION

The WHO estimates that one in 10 medical products found in low- and middle-income countries (LMICs) is either substandard or falsified.^1^ The high cost of chemotherapy drugs makes them attractive targets for falsification in countries of all income levels. For example, in 2012 the US Food and Drug Administration issued a recall of the drug Avastin (Genentech, San Francisco, CA).^2^ According to a report by the Reuters news service,^3^ empty bottles were taken out of a clinic’s dumpster and refilled with water, corn starch, and acetone.^4^ In addition to such deliberate falsification, improper storage of medications can lead to drug degradation and result in a medication of lower potency than expected.^5^

Demand is increasing for chemotherapy drugs in LMICs because of increasing efforts to diagnose cancer and innovations such as the collaboration between the American Cancer Society and the Clinton Health Access Initiative to provide lower prices on 16 chemotherapy drugs in six African countries.^6^ From 2012 to 2025, the cancer incidence is estimated to increase by 77%,^7^ from 14.1 million to 20 million new cases diagnosed annually, and most of those cases will be diagnosed in LMICs.^8,9^

The increase in demand for chemotherapy drugs will create new opportunities for unethical manufacturers and distributors, a pattern seen in other health interventions. For example, in the mid-2000s, global health organizations began an ambitious program to reduce malaria deaths by providing subsidized insecticide-treated bed nets, rapid diagnostic tests, and artemisinin combination therapy drugs. Unscrupulous manufacturers soon flooded markets with ineffective bed nets,^10^ poor-quality rapid diagnostic tests,^1^ and counterfeit artemisinin combination therapy drugs.^11-13^ Chemotherapy drugs are attractive targets for falsification because of their high selling prices and the high background mortality rate of patients with cancer, yet there is little post-market monitoring of chemotherapy agents,^5^ especially in LMICs. The US Pharmacopoeia has helped develop sustainable medicines quality monitoring programs in many countries in Africa, Asia, Eastern Europe, Latin America, and the Caribbean. However, the US Pharmacopoeia Medicines Quality Database, which contains more than 16,900 entries for anti-infective medicines, has no entries for chemotherapy or anticancer drugs. This reflects the lack of testing, rather than universal good quality. The WHO Global Surveillance and Monitoring System for substandard and falsified medical products obtains reports of poor-quality products from pharmaceutical companies and regulatory agencies. Between 2013 and 2017, 100 substandard or falsified chemotherapy drugs were reported from 19 WHO member states.^1^ One of these resulted in a Rapid Alert report.^14^

Currently, the most common problem reported for anticancer drugs is the absence of the active pharmaceutical ingredient (API). In January 2017, an investigation by the Mexican Secretary of Health reported that a state-run hospital in Veracruz, Mexico, had purchased falsified Avastin that contained 0% of the API in 2010 and 2011.^15^ Roche, the manufacturer on the packages, confirmed that these products were falsified and came from unauthorized distributors. At the hospital, 119 adults were treated with Avastin between 2010 and 2016 and could have been affected by this product. To ensure that patients receive effective treatment, the world scientific and drug regulatory community must be proactive in monitoring chemotherapy drug quality at all levels of the supply chain.

In low-resource settings, there are limited ways to test if an anticancer medication contains the active ingredient in the correct amount. Presence/absence of the active ingredient can be assessed by colorimetric methods,^16^ thin-layer chromatography (TLC),^17-22^ ultraviolet-visible spectroscopy,^18,23^ biosensors,^24-27^ and infrared spectroscopy or surface-enhanced Raman scattering (SERS).^28^ Quantification can be accomplished by using TLC, high-performance liquid chromatography (HPLC) or liquid chromatography/mass spectroscopy (LC-MS), ultraviolet-visible spectroscopy, biosensors, and SERS. Some weaknesses of these methods in developing-world settings include the need for analytic balances, glassware, instrumentation, and electrical power and the use of organic solvents or expensive consumable supplies. The Global Pharma Health Fund (Giessen, Germany)-Minilab, which is based on TLC, is commonly used as a screening tool for antimalarial, antimicrobial, antitubercular, antihelminthic, antiretroviral, and cardiovascular drugs.^29^ However, it does not include protocols for chemotherapy drugs.

This report describes a simple paper test card, the chemoPAD, that can detect the presence or absence of four common chemotherapy agents using the droplets left in vials after patients receive chemotherapy. The user does not need to handle chemicals other than the injectable drug and water. The card can monitor injectable methotrexate, doxorubicin, cisplatin, and oxaliplatin, which are included in the Clinton Health Access Initiative, are listed on the WHO List of Essential Medicines,^30^ and are widely used in LMICs. The card is intended as an early warning sign of gross falsification in a country’s supply chain, not as a replacement for conventional pharmaceutical analysis. It is designed for widespread implementation through an LMIC health care system with the aim of monitoring thousands of samples per year. Testing fresh vials of chemotherapy drugs in such numbers would be expensive and would limit patient access to the drugs, which is why the card is designed to use the drops that remain in the vial after patient treatments. If the chemoPAD results are suspicious, the pharmacist can switch to another brand of the drug while samples from the suspect brand are sent to the drug regulatory agency for a confirmatory assay. For a point of use device to succeed in this use scenario, it must be accurate, robust, and usable by oncology pharmacists and nurses; we evaluated the chemoPAD to see if it could meet these criteria.

**CONTEXT SUMMARY**

The chemoPAD is a new low cost, user-friendly technology for screening chemo-drug quality in low and middle income countries. This paper-based device uses color chemistry to visually test for the presence of four active pharmaceutical ingredients (API) found in chemotherapy drugs. The chemoPAD was tested in Black Lion Hospital in collaboration with Addis Ababa University in Ethiopia and the University of Notre Dame in South Bend Indiana. This device can be used by pharmacists in low and middle income countries to quickly screen four different cancer drugs for API content.

## RESULTS

The chemoPAD test card was designed to detect the presence or absence of cisplatin, doxorubicin, methotrexate, and oxaliplatin. After the test card was validated in the laboratory using blind samples prepared from US brands of the four drugs, samples of dosage forms were collected and screened in Ethiopia. These samples were then transported to the United States for HPLC analysis.

### Test Card Design

#### General considerations.

**Fig 1:**
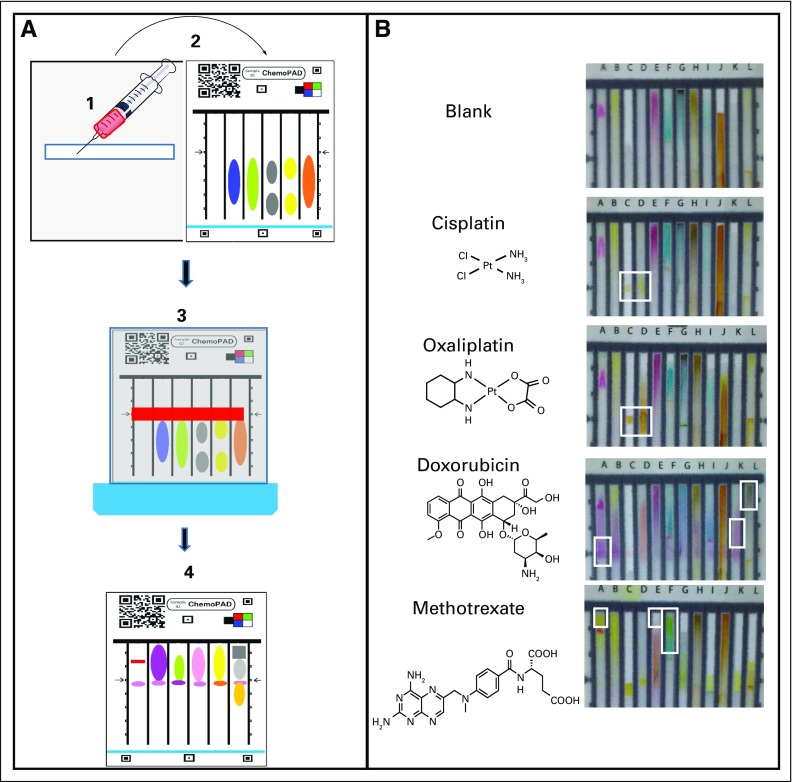
Schematic of chemoPAD usage. (A) (1) Apply approximately 65 μL of drug solution to test application strip; (2) fold over and press test application strip into chemical test region; (3) place in small dish of water for 3 minutes; and (4) remove card from water and take a picture to record color bar code. (B) The chemoPAD bar codes for cisplatin, oxaliplatin, doxorubicin, and methotrexate are shown; the white boxes indicate areas of interest for identification of each drug.

Work with chemotherapy drugs comes with several challenges. Injectable chemotherapy drugs typically come prepared in dilute solution, and—even in low concentrations—they are hazardous to handle because of their high toxicity. Other challenges are the scarcity of the medications and high cost of the medications. These challenges were addressed in the test card design (Fig 1A). The chemoPAD is covered with a plastic sheet that creates a physical barrier between the user and the drugs. The design incorporates a sample application strip made from absorbent paper, which the user can load by removing 65 μL from the vial with a syringe and applying it to the strip. The side of the card that contains the sample application strip is then folded over and pressed into the chemical testing portion of the card, so a few microliters of the sample are transferred to each of the 12 lanes. The device is placed into a small dish of water for approximately 3 minutes or until the water has reached the top of the 12 test lanes. The card is removed from the water dish and photographed to capture the test results. This picture can be compared with standard images to identify if the sample contains the expected API (Fig 1B).

#### Development of color tests used to detect chemotherapy agents.

The principle of operation of the paper analytic device (PAD) is that multiple chemical color tests are performed in parallel on the analyte. Twelve lanes are formed by printing hydrophobic barriers onto the paper, and the reagents for individual chemical color reactions are stored in dry form on separate lanes of the PAD. A few microliters of the analyte are deposited approximately two thirds of the way up each of the lanes by pressing the application strip into the lanes. When the bottom edge of the PAD is dipped into water, all of the tests are activated. Water climbs up the 12 lanes by capillary action and carries the reagents to the analyte. The reactions between the reagents and the analyte create a color bar code, which can be matched to bar codes from known good samples.

Several lanes are included on the chemoPAD to detect possible adulterants, fillers, or substitute APIs that might be included in a falsified chemotherapy product. These lanes include tests for nucleophiles, tertiary and primary amines, β-lactams, starch, and carbonates and a timer lane.^31,32^ Additional lanes were created to detect the APIs in the four targeted chemotherapy drugs.

#### Detection of cisplatin and oxaliplatin with stannous chloride.

Stannous chloride is a strong reductant that has been used as a color test to detect platinum compounds^33^ and as a TLC stain for cisplatin.^34^ Cisplatin and oxaliplatin react with it to form yellow products (λ_max_ = 475 nm; Data Supplement). We hypothesized that the color change was due to the formation of platinum/tin nanoparticles,^35^ which was confirmed via scanning electron microscopy/energy dispersive X-ray (Data Supplement). The reagents for the reaction are stored by allowing a solution of SnCl_2_ to dry in the designated lane of the PAD. The stannous chloride lane was stable for at least 4 weeks at room temperature and for 2 weeks at 35°C. Figure 1B shows the PAD bar codes expected from cisplatin and oxaliplatin dosage forms.

This lane test was found to be semiquantitative. It therefore is useful both to detect severely diluted drugs that have less than 50% of the stated API content and to distinguish between the common dosage forms of cisplatin and oxaliplatin, which are provided at different concentrations (1 mg/mL and 5 mg/mL, respectively).

#### Detection of doxorubicin by metal complexation.

Doxorubicin is a polyphenol that forms colored complexes with various divalent and trivalent transition metals. The chemoPAD already had several lanes that contained copper(II), nickel(II), and iron(III) for detection of possible substitute APIs.^31,32^ The copper(II) lane gave a reliable dark purple color signal with doxorubicin at concentrations typical of the dosage (2 mg/mL). Two new test lanes were added: lane G contained vanadium(IV) in the form of vanadyl sulfate, and lane K contained zinc(II). In the presence of a base (provided via a spot of 1 M of NaOH at the bottom of the lanes), these metals react with doxorubicin on the test card to form purple colors.

#### Detection of methotrexate via copper complexation.

Methotrexate contains several chelating nitrogens and forms a green complex with copper.^36^ Copper was present on the chemoPAD in lane F, which uses copper(II) and potassium carbonate to detect β-lactam groups.^31,32^ Methotrexate produces a green color in this lane that is distinct from the blue color of the copper ions and the dark green or black found with β-lactam antibiotics.

#### Threshold values for chemoPAD.

**Fig 2:**
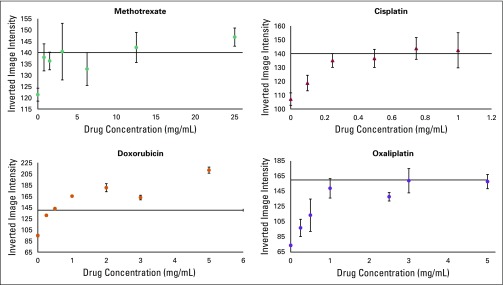
Plots that show the relationship between drug concentration and color quantified by ImageJ for the following tests: lane F, methotrexate; lane K, doxorubicin; lane D, cisplatin and oxaliplatin. For each drug, two data series are represented (closed *v* open circles), which show the cards produced and run on different days. The horizontal line represents the threshold chosen for each color response. Data points show the average of three test cards, and error bars represent standard deviation.

A total of eight lane tests respond to the four chemotherapy drugs. To determine the useful concentration range for each of the drugs, the most responsive lane was chosen for each drug. For cisplatin (1 mg/mL) and oxaliplatin (5 mg/mL), it was the stannous chloride test in lane D; for doxorubicin (2 mg/mL), the zinc test in lane K; and for methotrexate (25 mg/mL), the copper test in lane F. Color intensities were measured using ImageJ (National Institutes of Health, Bethesda, Maryland).^37^ Threshold values were set using the average color intensities observed for the typical dosage form concentration (Fig 2). The copper test in lane F and the zinc test in lane K exhibited all-or-nothing color responses at the concentrations used, so these tests for methotrexate and doxorubicin were purely qualitative. However, the stannous chloride test in lane D was able to distinguish dosage forms of cisplatin (1 mg/mL) and oxaliplatin (5 mg/mL) and showed promise for detection of grossly substandard cisplatin and oxaliplatin products (API content < 50%).

### Internal Validation Study for Chemotherapy Drugs

Drug solutions for the four APIs were made up at three different concentrations: 100%, 50%, and 0%. These samples were made from standard API and dissolved in either water (methotrexate), 0.1 M HCl (doxorubicin), 0.9% saline (cisplatin), or 25 mg/mL (lactose) to mimic common dosage formulations. The 100% level was set as the concentration found in the most commonly used dosage forms, and it was diluted to 50% with either saline solution (for cisplatin), lactose (for oxaliplatin), or water. The 0% solutions were made using either saline solution (cisplatin), lactose (oxaliplatin), or water with different colored dyes (yellow food coloring, doxycycline, or rifampicin) to emulate the color of doxorubicin or methotrexate. The samples were blinded, and each sample was run on two or more chemoPADs. The images of the cards were taken in a homemade lightbox with a cellphone camera. HPLC analysis was conducted for all blind samples. The HPLC results were compared with the chemoPAD results obtained from ImageJ analysis or from the evaluation of human readers (Tables 1 and 2, respectively). For methotrexate and doxorubicin, when three lanes were assessed, a majority-passes rule was followed. For example, if two of three lanes passed, then that sample was said to pass; if one of three passed, then that sample was said to fail. In the case of oxaliplatin, when two lanes were assessed, if either C or D passed, then the sample passed. For cisplatin, only lane D was assessed.

**Table 1:**
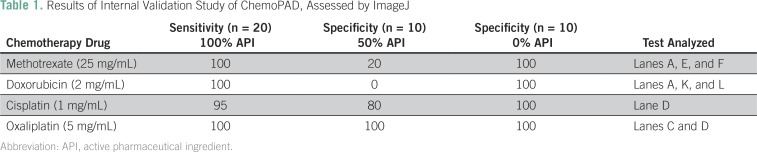
Results of Internal Validation Study of ChemoPAD, Assessed by ImageJ

**Table 2:**
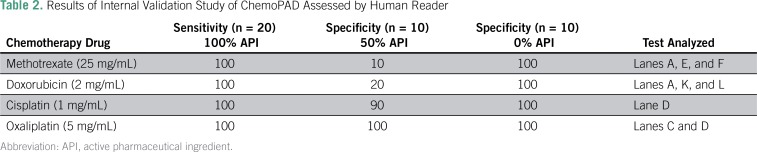
Results of Internal Validation Study of ChemoPAD Assessed by Human Reader

The chemoPAD had excellent sensitivity and specificity for detection of the 0% API samples. The 50% samples yielded mixed results; 50% cisplatin and oxaliplatin samples were well distinguished from the 100% samples, and specificities were 90% and 100%, respectively, for evaluation by a human reader and 80% and 100%, respectively, for evaluation by ImageJ analysis. However, the card was not able to detect the 50% (underdosed) samples of methotrexate and doxorubicin. To compare interuser results, a student was trained to use and read the chemoPAD. The newly trained student had the same overall reading results as the experienced user (Data Supplement).

### Evaluation of Blinded Cisplatin Dosage Forms in Ethiopia

Good (100% API) and bad (50% and 1% API) samples of cisplatin were created from a standard solution, and the reviewers were blinded to the solutions. A pharmacist and pharmacy professor at Black Lion tested the unknown samples using the chemoPAD and compared the results with a standard image. Two cards were run for each sample, and a third was run in the case of ambiguous results. A sensitivity of 100% (n = 3) and a specificity of 83.3% (n = 6) were obtained (Table 3). Only one sample was misidentified: a sample at 50% was incorrectly identified as 100% API.

**Table 3:**
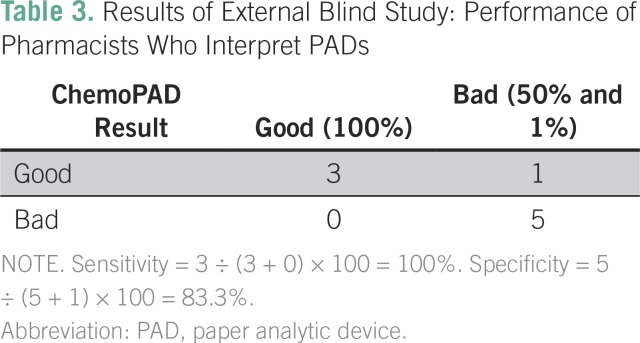
Results of External Blind Study: Performance of Pharmacists Who Interpret PADs

### Application of the chemoPAD to Post-Market Samples From Ethiopia

#### Sample collection.

The anticancer drugs for this study were collected from university teaching hospitals in Ethiopia, which included the following: Black Lion (Tikur Anbessa) specialized hospital, Addis Ababa; Ayder Referral Hospital, Mekelle, Tigray regional state; Hawassa University Teaching Hospital, Hawassa, Southern Nations and Nationalities and Peoples Regional State; and Jimma University Hospital, Jimma, Oromia regional state. Additional information about sample collection can be found in the Data Supplement. Multiple samples of each product were purchased so that one sample could be opened and tested by PAD, another could be sent for assay at the University of Notre Dame, and an unopened sample could be retained in Ethiopia in case follow-up with the regulatory agency was needed.

#### Screening and HPLC results.

All of the products were screened with the chemoPAD at Black Lion Hospital in Ethiopia. By visual comparison with standard images, and by computer image analysis of the PAD results, all samples passed. The chemoPAD predicted that the cisplatin and oxaliplatin products contained at least 50% of the stated API content. Because the doxorubicin and methotrexate tests were qualitative, the PAD results showed only that there was at least some of the correct API present in each product. The samples were brought to Notre Dame for confirmatory analysis, which was consistent with the predictions in each case (Table 4).

**Table 4:**
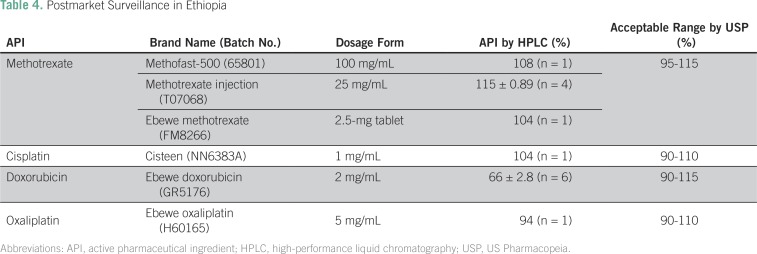
Postmarket Surveillance in Ethiopia

Although all of the products had the correct API present, doxorubicin was substandard and contained only 66% of the stated API; as a consequence, it was not identified by the chemoPAD, which is a qualitative tool for this drug. We explored possible reasons for this low concentration. The samples were transported by air during mild fall weather. They were unrefrigerated for 2 days during this transport and then stored for 1 month in a 4°C fridge. A stability mini-study was conducted to ensure that the drug did not degrade in transport from Ethiopia to Notre Dame. A bottle of doxorubicin was left out at room temperature but protected from light. The concentration remained stable for 3 weeks and then started to slowly degrade over the course of 5 months, and the concentration dropped from 1.3 mg/mL to 0.8 mg/mL (Data Supplement). This shows that the sample degradation from 100% to 66% over the course of 2 days at room temperature was unlikely. This substandard product was reported to the regulatory agency in Ethiopia.

## DISCUSSION

The chemoPAD is a simple and fast test for drug quality that only requires a small dish of water and a syringe to apply liquid samples. The chemoPAD can be used as a qualitative device to detect the presence of four APIs. Because of the toxicity of these drugs, this device could be used by health care practitioners who are already trained to handle chemotherapy drugs and have access to a fume hood or laminar flow hood used to compound such drugs into intravenous bags.

The chemoPAD could bridge the gap between analytic laboratories, regulatory agencies, and health care providers to monitor the quality of small-molecule chemotherapy agents. For example, last year, the WHO Rapid Alert system issued a warning that a falsified version of Sutent (sunitinib malate; Pfizer, New York, NY) had been seized by the National Drug Authority in Uganda. This small-molecule chemotherapy agent and the fake Avastin were both being distributed near various cancer treatment centers in Kampala, Uganda. The Pan-American Health Organization reported that 11 batches of falsified Sutent were found in Brazil in March 2018.^38^ A fast and simple field test could have helped health care providers identify these fake products more quickly.
